# Developments in spiritual care education in German - speaking countries

**DOI:** 10.1186/1472-6920-14-112

**Published:** 2014-06-05

**Authors:** Piret Paal, Traugott Roser, Eckhard Frick

**Affiliations:** 1 Spiritual Care, Department of Palliative Medicine at the Ludwig Maximilian University of Munich, Munich, Germany; 2 Faculty of Practical Theology at the Westfälische Wilhelms University of Münster, Münster, Germany

## Abstract

**Background:**

This article examines spiritual care training provided to healthcare professionals in Germany, Austria and Switzerland. The paper reveals the current extent of available training while defining the target group(s) and teaching aims. In addition to those, we will provide an analysis of delivered competencies, applied teaching and performance assessment methods.

**Methods:**

In 2013, an anonymous online survey was conducted among the members of the International Society for Health and Spiritual Care. The survey consisted of 10 questions and an open field for best practice advice. SPSS21 was used for statistical data analysis and the MAXQDA2007 for thematic content analysis.

**Results:**

33 participants participated in the survey. The main providers of spiritual care training are hospitals (36%, n = 18). 57% (n = 17) of spiritual care training forms part of palliative care education. 43% (n = 13) of spiritual care education is primarily bound to the Christian tradition. 36% (n = 11) of provided trainings have no direct association with any religious conviction. 64% (n = 19) of respondents admitted that they do not use any specific definition for spiritual care. 22% (n = 14) of available spiritual care education leads to some academic degree. 30% (n = 19) of training form part of an education programme leading to a formal qualification. Content analysis revealed that spiritual training for medical students, physicians in paediatrics, and chaplains take place only in the context of palliative care education. Courses provided for multidisciplinary team education may be part of palliative care training. Other themes, such as deep listening, compassionate presence, bedside spirituality or biographical work on the basis of logo-therapy, are discussed within the framework of spiritual care.

**Conclusions:**

Spiritual care is often approached as an integral part of grief management, communication/interaction training, palliative care, (medical) ethics, psychological or religious counselling or cultural competencies. Respondents point out the importance of competency based spiritual care education, practical training and maintaining the link between spiritual care education and clinical practice. Further elaboration on the specifics of spiritual care core competencies, teaching and performance assessment methods is needed.

## Background

Several studies have shown that there is an increasing demand for spiritual care training and a competency based spiritual care curriculum among health care professionals [1-7]. However, little is known about spiritual care training outside the UK and North America, neither do we know much about how to teach spiritual care effectively [8]. Discussion on the core competencies and central aims of spiritual care is ongoing. For example, a recent European guideline on palliative care core competencies states that the providers of palliative care should be able to meet patients’ spiritual needs, to raise and discuss spiritual/existential issues, demonstrate their individual understanding and reflective capacity, integrate spirituality in the care plan, handle issues with respect and support, and be conscious of the boundaries in terms of cultural taboos, values and choices [9]. Discussions in a European context, for example within the European Association for Palliative Care Task Force on spiritual care, have led to an understanding that in order to promote and develop the national and regional curricula in spirituality and health more information in terms of *what is going on* is needed [10].

In German-speaking countries, spirituality has gained an important place in comprehensive patient centred healthcare with increased interest among various health care professions during the past decade. Simultaneously, different training programmes have been introduced. So far, many developments in spiritual care education have been directly connected to palliative and end of life care education. Spirituality in healthcare, however, has gained standing in other fields of medicine, such as psychology, oncology, paediatrics, and intensive care. Such developments set a demand for solid knowledge about the requirements for spiritual care trainings, including setting explicit aims, pre-defining core competencies for different professions, as well as deciding on suitable teaching and performance assessment methods.

## Methods

The following article introduces the results of an explorative survey regarding spiritual care training conducted among the International Society for Health and Spirituality (IGGS) – members from Germany, Austria and Switzerland in early 2013. The overall aim of our online survey was to examine the current situation of spiritual care in undergraduate and graduate courses among the various healthcare professions in German-speaking countries. More explicitly we wanted to a) gain insight into organisation and structures, b) identify educational goals and core competencies, c) inquire about teaching and performance assessment tools, and d) learn about Best Practise. Findings should foster further developments in this rapidly growing field.

The anonymous online questionnaire was launched in February 2013. Invitations were sent out using the mailing list of IGGS members. A reminder was sent out in March and the survey was closed in April 2013. The survey consisted of 10 questions with multiple choice responses (in total 85 items) and an additional field for personal best practice advice or other comments. The survey combined multiple choice questions with more than one possible answer and open ended questions. The estimated time to fill out the questionnaire was 15 minutes. The SPSS21 was used for statistical data analysis and the MAXQDA2007 for thematic content analysis [11].

The ethics committee of Ludwig Maximilian University in Munich ruled (UE No 051–13) that ethical approval and consent were not required to conduct this study.

## Results

33 respondents participated in the survey. The IGGS, which has no denominational or religious affiliations, currently has 184 members: 139 from Germany, 11 from Austria, 28 from Switzerland and 6 from other countries. We addressed the IGGS members in German-speaking countries, accordingly, the response rate was 18.5%.

### Setting

The majority of spiritual care training is located in hospitals 36%, (n = 18). The survey indicates that 21% (n = 10) of spiritual care education is provided by universities: 6% (n = 3) faculty of theology, 11% (n = 5) medicine, 2% (n = 1) psychology, 2% (n = 1) interdisciplinary collaboration. In addition, 17% (n = 8) of trainings are delivered by different societies or associations, 17% (n = 8) by training/retreat centres, and 6% (n = 3) by hospice academies.

57% (n = 17) of spiritual care training are part of palliative care education provided in hospitals and medical faculties.

49% (n = 15) of spiritual care education is primarily bound to the Christian tradition, 6% (n = 2) to the Buddhist/Christian tradition and 6% (n = 2) of training approached spirituality from a multi-religious perspective. 36% (n = 11) of provided instruction have no direct association with any religion.

The survey demonstrates that 18% (n = 24) of spiritual care training are provided to nurses, 13% (n = 18) to social workers, 11% (n = 15) to chaplains, 11% (n = 15) to physicians working on wards, 8% (n = 11) to physiotherapists, 15% (n = 20) to medical students, 8% (n = 11) to theological students, and 10% (n = 13) to psychology students. Few courses are delivered to pharmacists 2% (n = 2), parents of sick children, general public or hospice volunteers 2% (n = 3), statutory management 1% (n = 1), and non-medical healers 15% (n = 1).

22% (n = 14) of available spiritual care education leads to some academic degree, 30% (n = 19) to certified qualifications. Seminars (12%, n = 8), lectures (22%, n = 14), practical exercises (9%, n = 6) or retreats and meditation (5%, n = 3) are part of some larger educational programme, such as palliative care, counselling psychology, or pastoral care. Additionally, spiritual care education may be provided as an internal training, individual supervision or as an event for special interest groups.

64% (n = 19) of respondents do not use any specific definition of spiritual care. 13% (n = 4) use the working definition proposed by EAPC Taskforce on Spiritual Care [12]. 3% (n = 1) use the definition of spiritual care provided by Roser and Frick [13]. One respondent (3%, n = 1) introduces various definitions during the training sessions. 17% (n = 5) provide their own definitions.

### Teaching aims and course duration

Content analysis revealed that spiritual training for medical students [4-12], physicians in paediatrics [38–41], and chaplains [42–44] belongs exclusively to the larger context of palliative care education. Courses provided for a multidisciplinary team may be part of palliative care training [22–26]. Other themes, such as deep listening [17], compassionate presence [16], bedside spirituality [21], or biographical work on the basis of logo-therapy [19], are discussed within the framework of spiritual care [13–21] (Table 1).

**Table 1:** Spiritual care training: aims and duration

**Target group**	**Course**	**Duration**	**Discipline**
Theology students	[1] Community internship	104 h	Theology
Psychology students	[2] Counselling psychology	2 years	Psychology
	[3] Colloquium on meditation practices, science, work and health	2 days	
Medical students	[4] Communication with cancer patients	60 min	Palliative care
	[5] Influence of stories and pictures	60 min	
	[6] The role of chaplaincy	90 min	
	[7] Grief	90 min	
	[8] Psychosocial-spiritual palliative care	144 h	
	[9] Spiritual care	2 × 45 min	
	[10] Spiritual care	2 × 45 min	
	[11] Change of mind-set and patient visits	40 × 45 min	
	[12] Spirituality and spiritual care	16 × 45 min	
Multidisciplinary team	[13] Spiritual assessment	180 min	Spiritual care
	[14] Providing spiritual support	2 × 45 min	
	[15] Spiritual care course	7 × 3 days	
	[16] Compassionate presence	22 × 45 min	
	[17] Deep listening	34 × 45 min	
	[18] Spiritual assessment	2 days	
	[19] Biographical work on the basis of logo- therapy (V. Frankl)	3 days	
	[20] Spiritual care course	7 × 2 days	
	[21] Bedside spirituality	7 × 45 min	
	[22] Solace	2 × 45 min	Palliative care
	[23] Quality management	2 × 45 min	
	[24] Providing spiritual support	2 × 45 min	
	[25] Spiritual assessment	60 min	
	[26] Palliative care	-	
Nurses	[27] Palliative care for nurses	-	Palliative care
	[28] Providing spiritual support	8 × 45min	Spiritual care
Physicians	[29] Spiritual care for beginners	2 × 135 min	Spiritual care
	[30] Leadership and spirituality	7 × 45 min	
	[31] Ethical issues in oncology	24 × 60 min	Oncology
	[32] Symposium of complementary care	1 per year	
	[33] Oncology and palliative care	-	
	[34] Ethical issues in palliative care	4 × 60 min	Palliative care
	[35] Terminal care in oncology	-	
	[36] Pilgrimage	5 days	
	[37] Palliative care	-	
	[38] Paediatric palliative medicine	12 × 45 min	Paediatric palliative care
	[39] Multi-faith spiritual care	2 × 45 min	
	[40] Spiritual care in Christian context	2 × 45 min	
	[41] Work with selected cases	2 × 45 min	
Chaplains	[42] Palliative care for chaplains	120 h	Palliative care
	Palliative care for chaplains	120 h + 20 h retreat	Palliative care
	Chaplaincy in intensive care units	80 × 45 min + 3 months practical work	Intensive care
General public, voluntary workers, family members	[43] Spiritual retreat	-	Spiritual care
	[44] Spirituality in hospice work	5 × 7 × 45 min	
	[45] Spirituality in hospice work	-	
	[46] Communication on the borders	3 × 45	
	[47] Healing grief	22 × 45 min	
	[48] Tibetan Buddhist teachings	3 days	
	[49] Parental evening	3 days	
	[50] Café: Life goes on	2 × 60 min	
	[51] Healing dance for orphaned mothers	-	
	[52] Healing painting for orphaned parents	-	
	[53] Seasonal letter	3 × per year	
	[54] Spiritual care, dementia and paintings	1 day	
	[55] Culturally sensitive spirituality in the care for the dying	16 × 45 min	
	[56] Spiritual care for people with dementia	3 × 45 min	
	[57] Rituals in the care for dying and grief management	8 × 45 min	
		[58] Spiritual care and chaplaincy: chances and possibilities	-

Spiritual care training: aims and duration.

### Core competencies

**Figure 1:**
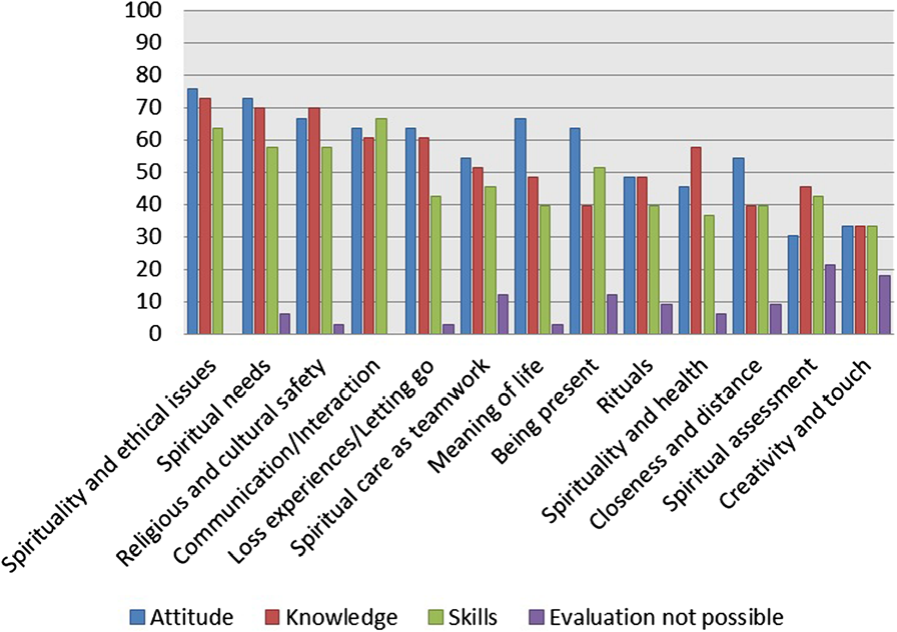
The frequency (%) of spiritual care competencies.

The questionnaire enclosed a list of spiritual care competencies designed on the basis of a focus group discussion on spiritual care education conducted in 2012. The competencies were divided into three sets: attitudes, knowledge, and skills (Figure 1). Participants were asked to evaluate the frequency of given competencies within their educational model. The results indicate that the essential themes concentrate on the attitudes and knowledge of spirituality and ethical issues, spiritual needs, religious and cultural sensitivity, and communication/interaction. In terms of skills, identification of spiritual needs, communication, and ethical counselling were given priority.

### Teaching methods

Table 2 provides an explorative overview of methods in teaching spiritual care. There appears to be an appropriate distribution between providing knowledge, developing attitudes and working on participants personal and professional skills. In providing knowledge, lectures [a], PowerPoint presentations [b], journal clubs [c], discussion rounds [I, m], and individual conversations with mentors [g] are used. Attitudes are fostered by visits to patient care units [l], watching films [j], or discussing provided readings [k]. Practical exercises encompass the development of personal and professional skills, such as self-exploration [q], meditation [r] and self-care exercises [u], as well as theme-centred interaction (TCI) [t], case or ritual reviews [o, p] and spiritual [x] or biographical assessments [w].

**Table 2:** Teaching methods

	**Teaching method**	**Teaching aim**
[a]	Lecture (including external lecturers)	Knowledge
[b]	PowerPoint presentation	Knowledge
[c]	Journal club	Knowledge
[d]	Writing exercises	Knowledge
[e]	Presentations	Knowledge
[f]	Music/Songs	Knowledge/Attitude
[g]	Individual conversations	Knowledge/Attitude
[h]	Spiritual impulses	Knowledge/Attitude
[i]	Group discussion/Panel discussion/Small group discussion	Knowledge/Attitude
[j]	Videos/Films/Pictures/Symbols	Knowledge/Attitude
[k]	Work with biblical/interreligious texts, other readings	Knowledge/Attitude
[l]	Visiting patients in patient care units	Knowledge/Attitude
[m]	Role plays	Knowledge/Skills
[n]	Sharing personal experiences towards implementing learned contents in daily ward work	Knowledge/Skills
[o]	Case review	Skills
[p]	Ritual review	Skills
[q]	Interactive self-exploration	Skills
[r]	Meditation/Bodily exercises/(Group) Prayer	Skills
[s]	Practical exercises (alone, with partner, in small groups)	Skills
[t]	Theme-centred interaction (TCI) according to Ruth Cohn	Skills
[u]	Self-care exercises	Skills
[v]	Interview protocol	Skills
[w]	Biographical assessment	Skills
[x]	Spiritual assessment	Skills

Teaching methods.

### Performance assessment

The results indicate that 47% of provided spiritual care courses have no routine performance assessment to control the improvement of participants’ attitudes, knowledge and skills. Of the respondents, 21% disclosed having oral, 24% written and 8% practical performance assessment. Specialised spiritual care training programmes encompass degree work, such as final thesis (10–20 pages), final presentation or a case/ritual review.

### Respondents’ objectives for good practice

In the open-ended questions of our study, issues raised by the participants included requests to stakeholders, commencement of meaningful spiritual care training programmes and the specific role of pastoral care.

In terms of grievances, respondents called for governmental support and recognition in order to promote spiritual care and spiritual care training, pointing out a lack of official endorsement. While respondents generally agreed that there is an urgent need for spiritual care training, they voiced their impression that spiritual care is overlooked even within palliative trainings. Regarding spiritual care education, respondents would welcome a greater allotment of time for presenting the meaning of spirituality in healthcare, which in turn would promote issues such as spirituality, spiritual care, palliative care, clinical bioethics, grief counselling, and patient-doctor communication.

Regarding the teaching methods, in the perception of respondents, there are presently not enough satisfactory curricula available. Training formats that are well evaluated include retreats and meditation courses.

The participants surveyed in our study viewed a focus on individual spirituality as essential. They called for caution in the assessment and gaining of access to individual spirituality during training, as an overreach regarding individual spirituality would run contrary to the principles taught there. They stressed that in every situation, it is important to remain open-minded and creative.

In terms of educators’ competencies, the respondents pointed out the importance of involving experts who have field experience in providing spiritual care. Introducing professionals with diverse understanding of spirituality and religious backgrounds was viewed as equally important.

Respondents insisted that pastoral care should not be abandoned, as it provides deep knowledge about religion and human religiosity. They also warned that replacing healthcare chaplains with voluntary workers is and will be highly problematic. In case such a replacement proves unavoidable, appropriate spiritual care trainings would be of major significance, they stated. In this regard, respondents called upon government, church, and health care institutions to collaborate.

The participants from Germany indicated that the current strict understanding and handling of clergy-patient confidentiality, particularly regarding the documentation of patient’s spiritual needs, is viewed as ill-fated. Finally, current pastoral care tends to be exclusively Christian, which again may lead to undesirable outcomes in the context of assessing spirituality and providing spiritual care.

## Discussion

Our survey revealed several important aspects regarding the current state of spiritual care education. First of all, besides several structured trainings for undergraduate and graduate healthcare professionals, healthcare institutions provide a variety of spiritual care courses to volunteers, general public and family members without recognisable profile. The content and aims of training methods vary considerably. On the one hand, this reflects the lack of consensus regarding the matters of spirituality and spiritual care in healthcare. On the other hand, it suggests that different target groups may have different needs and aims when integrating spirituality and spiritual care in their educational models.

For medical undergraduates spirituality and spiritual care is provided as an integral part of palliative care curriculum. The same applies for paediatricians working on wards. Chaplains, on the other hand, become acquainted with spiritual care due to palliative or intensive core curricula and theological students due to community internship. Although the results indicated that 18% of spiritual care training is provided to nurses, content analysis revealed that a great number of spiritual care courses are provided as staff training to multidisciplinary teams. This is particularly beneficial in terms of approaching spirituality as teamwork when integrating spiritual care into the care plan [14].

Spiritual care courses provided for health care professionals tackled the following themes: providing spiritual support, spiritual care for beginners, spiritual assessment, compassionate presence, deep listening, biographical work and bedside spirituality as well as leadership and spirituality. More frequently, however, spiritual care is approached as an integral part of some other subject, such as religious and cultural sensitivity, grief counselling, communication/interaction training, clinical bioethics, complementary care or psychological or religious counselling. This might become problematic when a practical application of spiritual care is needed. Therefore, it is essential to introduce assessment tools and intervention models that help to identify spiritual distress and take care of a patient according to his or her preferences. This indicates an urgent need for competency-based spiritual care education that involves an adequate amount of practical training.

Openness and intensive self-assessment is expected from participants in spiritual care training. Talking about personal spirituality is often perceived as a taboo [15] and therefore meditation courses as well as retreats appear particularly suitable for tackling the meaning of spirituality from a personal point of view. All spiritual care providers should be able to identify their own spiritual needs [16,17].

In order to introduce the meaning of spirituality in healthcare, participants use different readings, visual and audio images, and other training methods that lead to self-exploration or group discussions. Practical tasks involve role plays, theme-centred interaction, case or ritual reviews, discussion of verbatim interaction transcripts, and biographical or spiritual assessments. The courses rarely involve visits to patient care units or real life bedside spiritual care training. This reveals an unfortunate inconsistency between teaching methods that raise awareness and educational aims that should be oriented more towards practice.

The link between spiritual care education and practical work is important, involvement of educators in field experience is essential. Furthermore, educators should be tolerant of diverse interpretations of spirituality and have a broad understanding of spirituality deriving from different religious contexts.

The survey revealed an insufficiency in performance assessment. To explore the advantages in skills, knowledge, and attitudes regarding spirituality and spiritual care, it would be important to integrate appropriate performance assessment methods that point out if the educational aims are met.

### Implications for practice

The first steps in planning spiritual care training or educational programme should involve considerations regarding what the overall framework for an education programme leading to a formal qualification. This involves: defining the institutional discourse, extent of the training, and the target group. The second question should inquire about reasons for incorporating spiritual care in this particular discourse. In case standardised clinical methods to assess spiritual distress and to provide spiritual care and healing are available or pre-defined, these should be integrated in educational models as preliminary training aims. If not, defining core competencies is essential. One should have in mind that the core competencies may be different even within the same discourse, for example, placing different expectations on healthcare chaplains working in palliative care units or intensive care units, not to mention those providing community services. A similar requirement applies to psychologists, social workers, physicians, nurses and other healthcare providers.

The second opportunity is to approach spiritual care as a team assignment. In that case retreats and spiritual care courses involving self-assessments and self-care may be beneficial. Additionally, trainings for teams should incorporate practical assignments with appropriate follow-ups, which guarantees the implementation of spiritual care and which help to take care of caregiver’s spiritual needs if needed.

The third important task is to consider the connection between teaching aims and teaching methods. In undergraduate education the teaching aims may involve intensive work with different concepts and definitions. However, in case the final aim is comprehensive patient care, the spiritual care training should involve practical assignments, such as spiritual assessment, compassionate presence, deep listening, biographical work or bedside spirituality work.

Finally, after defining the overall framework and educational aims as well as selecting appropriate teaching methods, it is important to decide on appropriate performance assessment methods. Performance assessment results and outcomes may be good indicators in terms of meeting the aim of the training.

### Limitations

With our pilot survey we addressed the members of the IGGS from Germany, Austria and Switzerland. The number of responses (33) and the calculated response rate of 18.5% may appear rather low. Nevertheless, it is important to understand that teaching spiritual care is in embryonic status in all German speaking-countries. Thus, we estimate that our survey succeeded to cover up to 75% of current spiritual care training provided to healthcare professionals.

The pilot survey did not include any information about respondents’ professional or individual background. However, participants’ personal background and self-estimated competencies could be interesting aspects for analysing the framework of spiritual care training and competencies delivered. Then again, available data indicates that respondents are simultaneously involved in different educational settings (university, hospice academy, etc.), which means that their roles and tasks as their teaching aims are dependent on the institutional setting. This leads to a suggestion for further surveys, namely that the institutional setting and target group(s) should be carefully pre-distinguished to provide a more sufficient overview.

## Conclusions

This article provides an overview about spiritual care education in Germany, Austria and Switzerland. A debate is going on in spiritual care education, but to date the discussion regarding the core competencies, teaching methods and sufficient performance assessment has reached no consensus. The results indicate that spiritual care education, particularly when provided for professionals working on wards, should become more practical, and involve work with patients under a follow-up mentoring programme and spiritual supervision. In undergraduate education spiritual care should be approached as an independent subject with well-defined aims. The article provides a list of objectives for planning successful spiritual care training. However, further elaboration on the specifics of spiritual care core competencies regarding different disciplines and discourses is obligatory.

## Competing interests

The authors have no significant competing financial, professional or personal interests that might have influenced the performance or presentation of the work described in this article.

## Authors’ contributions

PP and TR initiated the survey. PP carried out the statistical and qualitative analysis, drafted the manuscript, and did the scientific writing. TR and EF participated in the design of the study and helped to draft the manuscript. All authors read and approved the final manuscript.

## Pre-publication history

The pre-publication history for this paper can be accessed here:

http://www.biomedcentral.com/1472-6920/14/112/prepub
